# In vivo characterization of key iridoid biosynthesis pathway genes in catnip (*Nepeta cataria*)

**DOI:** 10.1007/s00425-022-04012-z

**Published:** 2022-10-12

**Authors:** Lira Palmer, Ling Chuang, Marlen Siegmund, Maritta Kunert, Kotaro Yamamoto, Prashant Sonawane, Sarah E. O’Connor

**Affiliations:** 1 Department of Natural Product Biosynthesis, Max Planck Institute for Chemical Ecology, 07743 Jena, Germany; 2 Institute of Botany, Leibniz University Hannover, 30167 Hannover, Germany; 3 School of Science, Association of International Arts and Science, Yokohama City University, 22-2 Seto, Kanazawa-ku, Yokohama, 236-0027 Japan

**Keywords:** Biosynthesis, Catnip, Iridoid, Metabolism, Natural product, Nepeta, Nepetalactone, Pathway, Virus-induced gene silencing (VIGS)

## Abstract

**Main conclusion:**

Using virus-induced gene silencing, we demonstrated that the enzymes GES, ISY, and MLPL are responsible for nepetalactone biosynthesis in *Nepeta cataria*.

**Abstract:**

Nepetalactone is the main iridoid that is found in the *Nepeta* genus and is well-known for its psychoactive effect on house cats. Moreover, there is a burgeoning interest into the effect of nepetalactone on insects. Although the enzymes for nepetalactone biosynthesis have been biochemically assayed in vitro*,* validation of the role that these enzymes have *in planta* has not been demonstrated. Virus-induced gene silencing (VIGS) is a silencing method that relies on transient transformation and is an approach that has been particularly successful when applied to a variety of non-model plants. Here, we use a recently designed visual-marker dependent VIGS system to demonstrate that the nepetalactone biosynthetic enzymes GES, ISY, and MLPL impact nepetalactone biosynthesis in *Nepeta cataria*.

**Supplementary Information:**

The online version contains supplementary material available at 10.1007/s00425-022-04012-z.

## Introduction

Species belonging to the Nepetoideae sub-family of mint (Lamiaceae) are well-known as terpene super producers (Mint Evolutionary Genomics Consortium 2018). Numerous members of this sub-family, including lavender, basil, and rosemary, produce terpenes that are important to the flavors and fragrance industries. Iridoids are a specialized class of monoterpene that is widely produced in the Lamiaceae. However, within the Nepetoideae sub-family, only the *Nepeta* genus produces iridoids. Nepetalactone is the main iridoid that is found in the *Nepeta* genus (McElvain et al. 1941; Formisano et al. 2011; Mint Evolutionary Genomics Consortium 2018; Sherden et al. 2018), and is well-known for its psychoactive effect on house cats (*Felis catus*) and other members of the Felidae family (Bol et al. 2017; Uenoyama et al. 2021). Moreover, there is a burgeoning interest into the effect of nepetalactone on insects such as mosquitoes and aphids (Dobler et al. 2011; Reichert et al. 2019; Melo et al. 2021; Uenoyama et al. 2021, 2022).

**Fig. 1:**
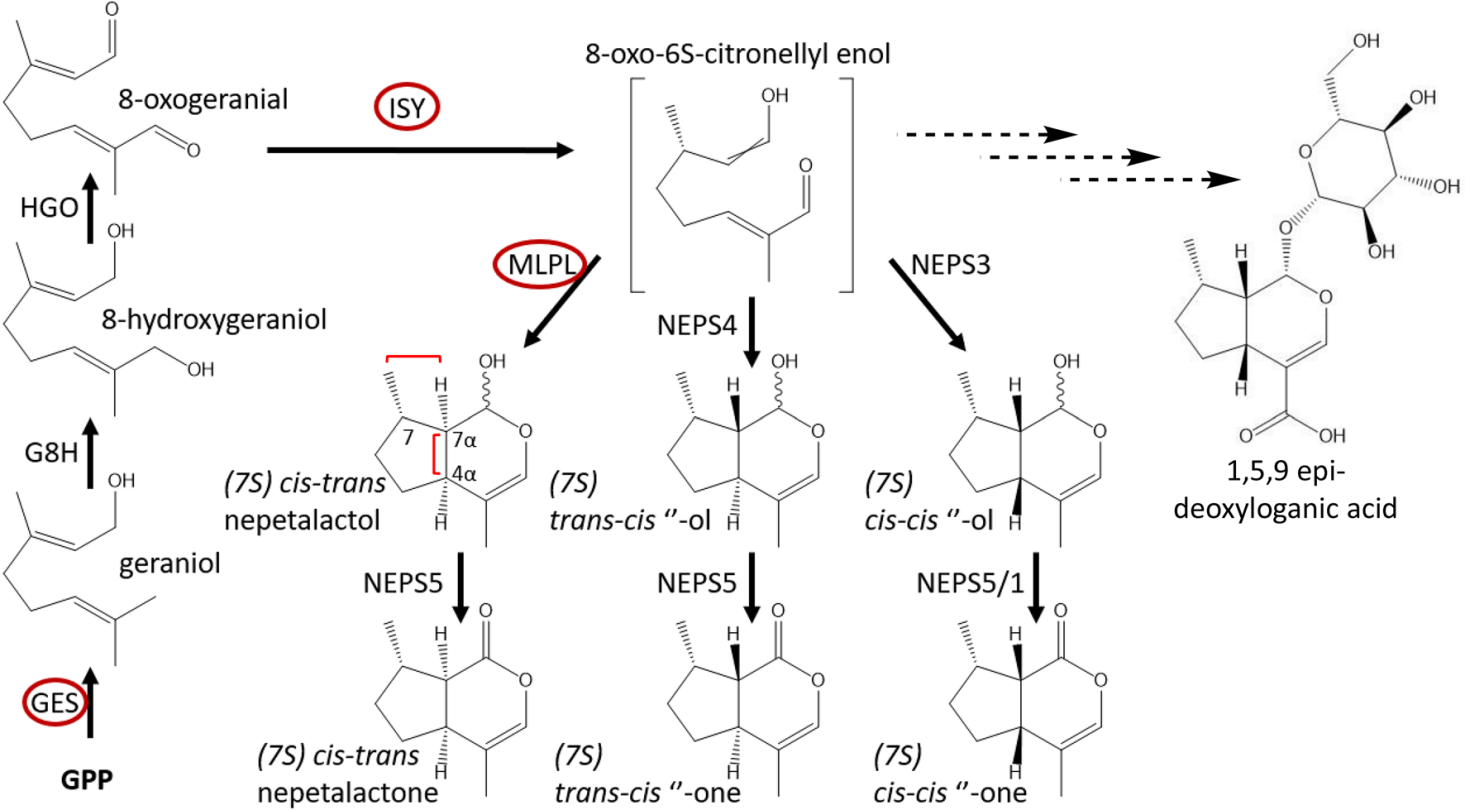
Iridoid biosynthesis pathway. A schematic of the *N. cataria* iridoid pathway based on in vitro studies. Enzymes in a red outline were targeted for in vivo silencing in this study. Key stereoisomeric carbons have been highlighted with their respective numbering

Nepetalactone biosynthesis in *Nepeta* begins, like all monoterpenes, with geranyl pyrophosphate (GPP), which is produced from the MEP pathway. GPP is converted to geraniol by the specialized terpene synthase, geraniol synthase (GES) (Simkin et al. 2013) (Fig. 1). Geraniol is hydroxylated by the CYP76 class II cytochrome P450 geraniol 8-hydroxylase (G8H) (Höfer et al. 2013; Miettinen et al. 2014; Parage et al. 2016; Lichman et al. 2020) and then further oxidized by the oxidoreductase hydroxygeraniol oxidase (HGO) (Miettinen et al. 2014; Krithika et al. 2015) to produce 8-oxogeranial. The *Nepeta* spp. homologues of GES, G8H, and HGO are similar to those identified in other iridoid producing plants (Lichman et al. 2020). The enzyme iridoid synthase (ISY) then performs a 1,4 reduction of 8-oxogeranial to yield a reactive intermediate (8-oxocitronellyl enol) that can then spontaneously cyclize to form predominantly the *cis,trans* stereoisomer of nepetalactol (Geu-flores et al. 2012; Miettinen et al. 2014; Kries et al. 2017; Nguyen and O’Connor 2020). Although ISY is found ubiquitously in all iridoid producing plants (Nguyen and O’Connor 2020), a detailed phylogenetic analysis (Mint Evolutionary Genomics Consortium 2018) suggests that ISY was lost in the Nepetoideae sub-family, and then re-evolved independently in the *Nepeta* genus from progesterone-5-beta reductase (P5βR). Thus, *Nepeta* ISY has a distinct catalytic active site compared to the ISY found in other iridoid producing plants (Sherden et al. 2018). Moreover, the presence of three stereochemical isomers of nepetalactone in *Nepeta* (*(7S)-cis,trans (4aS, 7aR)*, *(7S)-trans,cis (4aS, 7aS)* and *(7S)-cis,cis (4aR, 7aS*) led to the discovery that, although the ISY product 8-oxo-6*S*-citronellyl enol can non-enzymatically cyclize to the (*S*)-*cis,trans* nepetalactol stereoisomer, cyclization *in planta* appears to be assisted by dedicated enzymes that control cyclization to form specific nepetalactol stereoisomers (Fig. 1) (Sherden et al. 2018; Lichman et al. 2019). Three stereoselective cyclases were recently discovered in the trichomes of *Nepeta cataria* and *Nepeta mussinii* (Lichman et al. 2019, 2020). The biochemical activity of these enzymes showed that short-chain alcohol dehydrogenase like (SDR-like) enzymes NEPS3 and NEPS4 catalyse cyclization of 8-oxocitronellyl enol to the (*S*)-*cis,cis* and (*S*)-*trans,cis* nepetalactol isomers. A major-latex protein-like (MLPL) enzyme yields the (*S*)-*cis,trans* isomer (Lichman et al. 2020). NEPS like enzymes, NEPS5 and NEPS1, then oxidize these nepetalactols into nepetalactone (Lichman et al. 2019, 2020) (Fig. 1). In addition to the volatile nepetalactones produced in the pathway presented in Fig. 1, glycosylated iridoids have been reported in various *Nepeta* spp. (Murai et al. 1984; Formisano et al. 2011; Lichman et al. 2020), such as 1,5,9 epi-deoxyloganic acid (Fig. 1). However, the genes involved in the production of these glycosylated iridoids have not been identified.

NEPS and MLPL enzymes do not play any known role in iridoid pathways in other iridoid producing plants, and as described above, ISY from *Nepeta* spp. has a distinct evolutionary lineage. Although these *Nepeta* enzymes have been biochemically assayed in vitro (Sherden et al. 2018; Lichman et al. 2019, 2020), validation of the role that these enzymes have *in planta* has not been demonstrated. Virus-induced gene silencing (VIGS) is a silencing method that relies on transient transformation and is an approach has been particularly successful when applied to a variety of non-model plants (Lange et al. 2013). Viral sequences from known plant-infecting viruses are used to trigger the post-transcriptional gene silencing system of the plant, which leads to the production of siRNA transcripts. If short (150–350 bp) homologous sequences of the endogenous gene of interest are included in the vector, then the post-transcriptional gene silencing machinery inhibits translation of the gene of interest (Purkayastha and Dasgupta 2009). The vector system adapted from tobacco rattle virus TRV (pTRV1 and pTRV2) has been used successfully in a wide range of dicot plants (Velásquez et al. 2009; Liscombe and Connor 2011; Preston et al. 2014; Misra et al. 2017; Xu et al. 2018; Koudounas et al. 2020). Although VIGS has not been widely reported for plants in the Lamiaceae family, TRV has been successfully used for VIGS in plant families within the Lamiales order (Preston et al. 2014; Koudounas et al. 2020) and successfully used to study triterpene biosynthesis in the sweet basil (*Ocimum basilicum*), a member of the Nepetoideae sub-family (Misra et al. 2017).

A functional genomics tool such as VIGS can be used to deconvolute the role of enzymes in vivo. In this study, we use a recently designed visual-marker dependent VIGS system (Palmer and O’Connor 2020; Yamamoto et al. 2021) to validate the function of GES, ISY, and MLPL in *N. cataria*. Although the limitations of VIGS prevented a full analysis of the effects of silencing these enzymes, we nevertheless demonstrated that these genes contribute to nepetalactone biosynthesis in *N. cataria*.

## Materials and methods

### Plant growth conditions and propagation method

*Nepeta cataria* L. cuttings were taken from plants growing in a growth chamber with 15.5 h of full light (23 °C), 30 min of dusk and 30 min of dawn conditions, and 7.5 h of night (21 °C). Humidity was kept at 50%. Cuttings were taken to include 2–3 nodes. The bottom node was removed from leaves and inserted into water until rooted, about 1–2 weeks. Rooted cuttings were transplanted to a soil mix of 250 L of Klasmann TS1 (Klasmann-Deilmann, Geeste, Germany), 70 L of Klasmann Tonnubstrat and 34.5 L of Raiffeisen Baustoffe (Planta Düngemittel GmbH, Regenstauf, Germany) sand (0.7–1.2 mm). Plants were fertilized once a week with 0.1% Ferty 3 (Planta Düngemittel GmbH) and watered as necessary.

### Vector design

Sequences were obtained from *ChlH* (359 bp), *GES* (300 bp), *ISY* (451), and *MLPL* (309 bp). Inserts from cDNA were amplified using Invitrogen Platinum Superfi polymerase. Primers for each reaction can be found in primer list (Table S1). The *ChlH* fragment was cloned into the BamHI, XhoI site, and the *GES*, *ISY*, and *MLPL* fragments were cloned into the EcoRI site. Base pTRV-*ChlH* vectors were constructed via a ligation reaction.

### Virus-induced gene silencing

Electrocompetent *A. tumefaciens* strain GV3101 cells (50 μL) were transformed with 100 ng of plasmid DNA using electroporation and then plated on LB agar containing 50 mg/L of kanamycin, 50 mg/L of gentamycin, and 50 mg/L of rifampicin. Plates were incubated at 28 °C for 2 days. *A. tumefaciens* cultures for pTRV1, pTRV2, pTRV2-*ChlH*, pTRV2-*ChlH*-insert were grown in 50 mL LB cultures containing 50 mg/L of kanamycin, 50 mg/L of gentamycin and 50 mg/L of rifampicin, 10 mM MES buffer (pH 5.8) and 200 μM of acetosyringone for 24 h at 28 °C shaking at 200 rpm. Cultures were centrifuged for 15 min at 3500 g, the supernatant discarded and resuspended in 10 mL of fresh infiltration buffer (consisting of 10 mM of MES at pH 5.8, 10 mM of MgCl_2_ and 200 μM of acetosyringone). The OD_600_ was adjusted to 1. Cultures of pTRV1 *A. tumefaciens* were mixed with each individual pTRV2 *A. tumefaciens* cultures in a 1:1 ratio. Cultures were incubated in the dark for 3–4 h at room temperature with gentle rocking. Cultures were then centrifuged at 3500 g for 15 min, the supernatant was discarded and the pellet resuspended in 1 mL of fresh infiltration buffer. Plants for VIGS infection were cut down to 2–3 aerial nodes to encourage new leaf growth. A sterilized toothpick was dipped into the 1 mL cultures and used to wound the stem near the nodes 2–3 times, and finally the wounds were rubbed using the toothpick with more culture.

### VIGS tissue harvesting for metabolite analyses

Leaf tissue from pTRV2 empty vector and pTRV2-*GES* infected plants was collected by collecting a full leaf near the infection site. For plants infected with pTRV2-*ChlH* and pTRV2-*ChlH*-insert, only individuals with the photobleached phenotype were collected. Affected tissues was collected by cutting out the photobleached area from the green tissue and pooling together all affected leaf tissue from one individual. *N. cataria* tissue was flash frozen and pulverized with a Qiagen TissueLyser II at 25 Hz for 30 s twice. Ice-cold methanol containing the internal standards (200 μM in Suppl. Fig. S1a–b, or 50 μM) camphor and harpagoside (10 μM) was added to the powdered *N. cataria* tissue in a 20:1 (MeOH μL: tissue mg) ratio and sonicated at room temperature for 15 min. The mix was centrifuged on a table-top centrifuge at room temperature and at top speed (14,600*g*) for 10 min. The methanol extract was transferred to a new 1.5 mL tube. An aliquot was taken for LC–MS analysis, leaving 400 μL of the MeOH extract. 400 μL of hexane was added, and the mix was vortexed for 2 min, centrifuged at top speed for 30 s and the hexane layer was transferred to a solid phase extraction (SPE) column. The hexane layer was passed through the column and discarded. Then 400 μL of 20:80 EtAc:Hex (ethyl acetate: hexane) was added to the SPE column to collect the nepetalactones, collected for GC–MS analysis.

### Gas chromatography mass spectrometry and analyses

Samples were injected in split mode (1 μL, split ratio 10:1) at an inlet temperature of 230 °C on a Thermofisher Trace1310-ISQLT GC–MS at a MS transfer line temperature of 280 °C, ion source temperature of 250 °C and a CTC Analytics GC PAL autosampler. Separation was performed on a Zebron ZB5-HT-INFERNO column (5% phenyl-arylene and 95% dimethylpolysiloxane; length: 30 m; diameter: 250 μm; film thickness: 0.1 μm) with guard column (5 m). Helium was used as mobile phase at a constant flow rate of 1.1 mL/min. Two temperature runs were used for detection.

As shown in Fig. 3, after an initial temperature at 50 °C, the column temperature was increased to 170 °C at a rate of 10 K/min, then another increase to 280 °C at 50 K/min and maintained for 4 min. A solvent delay of 5 min was allowed before collecting MS spectra at electron ionization of 70 eV.

As shown in Suppl. Fig. S1, after an initial temperature at 60 °C, the column temperature was increased to 100 °C at a rate of 20 K/min, then to 160 °C at 2 K/min, then another increase to 280 °C at 120 K/min, and maintained for 4 min. A solvent delay of 5 min was allowed before collecting MS spectra at electron ionization of 70 eV.

Chemically characterized standards were used to identify compounds by retention time and electron impact spectra. Peaks of interest were obtained by the addition of the intensities at each time point and integrating the peak area according to a user input timeframe based on the file TIC. Nepetalactone peak areas were divided by the camphor peak area to obtain the normalized peak area. A pairwise *t* test with a Bonferroni adjustment was applied to obtain *p* values.

### RNA extraction and cDNA generation

Ground tissue powder was weighed out in portions of 25–35 mg of tissue into cooled 1.5 mL tubes. RNA was extracted using the Qiagen^®^ RNeasy PowerPlant Kit. Tissue was extracted according to kit instructions, with a final incubation on the filter membrane of 10 min. RNA concentration and A280/A260 and A280/A230 ratios were assessed using a Nanophotometer N60 and resolved on a 2% agarose gel. RNA samples of 1500 ng were treated with Sigma Amplification grade Dnase I kit and then the Thermo Fisher Scientific Applied Biosystems High-capacity cDNA reverse transcription kit was then immediately used to carry out the retro-transcription reaction.

### qPCR

Primers for qPCR analysis were designed using the CDS of the target gene in the NCBI primer design tool. The primers were restricted to replicate a product from 70 to 200 bp and to have a melting temperature of 60 °C. The cDNA generated from the RT reaction was diluted 2×. Amplification by qPCR was carried out using Agilent Brilliant II SYBR^®^ master mix. Each gene targeted had three technical replicates and three biological replicates. To measure gene expression, each target gene was paired to the housekeeping gene *UBI9*. This reaction was then placed in a Bio-Rad CFX96 Optical Reaction Module. The $2^{{ - \Delta \Delta C_{{\text{t}}} }}$ was used to measure gene expression.

### LC–MS method

For metabolite analysis, UPLC/MS was performed using an Impact II qTOF mass spectrometer (Bruker, Billerica, MA, USA) coupled to an Elute UPLC (Bruker) chromatographic system. Chromatographic separation was carried out on a Phenomenex Kinetex column XB-C18 (100 × 2.10 mm 2.6 μm particle size) kept at 40 °C and the binary solvent system consisted of solvent A (H_2_O + 0.1% formic acid) and solvent B (acetonitrile + 0.1% formic acid). Flow rate was 600 μL/min. The column was equilibrated with 99% A and 1% B. During the first minute of chromatography, solvent B reached 5%. Then a linear gradient from 5% B to 40% B in 5 min allowed the separation of the compounds of interest. The column was then washed at 100% B for 1.5 min and re-equilibrated to 1% B. Injection volume was 2 μL. Mass spectrometry was performed in negative ion mode with a scan range *m/z* 100–1000. The mass spectrometer was calibrated using sodium formate. The source settings were the following: capillary voltage 3.5 kV, nebulizer 2.5 Bar, dry gas 11.0 L/min, dry temperature 250 °C. Data analysis was performed using the Bruker Data Analysis Metaboscape (Version 2021-B) software.

## Results

### A VIGS visual marker aids in precise tissue selection

**Fig. 2:**
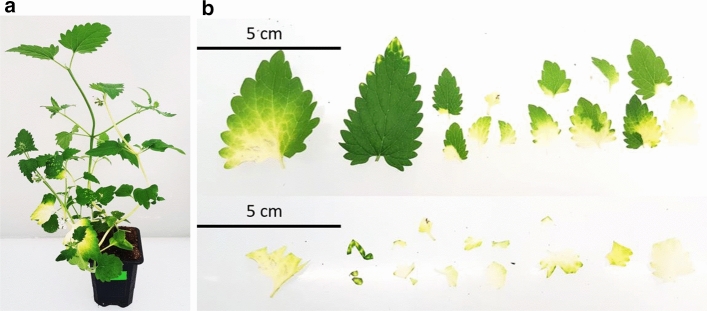
Representative *Nepeta cataria* plants subjected to VIGS*.* **a** A representative plant subjected to *ChlH* VIGS that exhibits the expected photobleaching, 4 weeks after infection. **b** An example of sample collection excising photobleached leaves. Top panel shows all the photobleached leaves of a single plant and the bottom panel shows the same leaves with only the photobleached tissue remaining

We previously established a method for VIGS in *N. cataria* cuttings (Palmer and O’Connor 2020) using the pTRV2 vector system. The gene for magnesium chelatase subunit H (*ChlH*), which is involved in chlorophyll biosynthesis, was targeted for silencing to provide a visual indicator of success in silencing. In this study we implemented a different method, similar to the method previously reported in Liu et al. (2002). Although this method modestly improved the efficiency of VIGS from an average of 25–32% of individuals as evidenced by the presentation of photobleaching in an experimental set-up (Suppl. Fig. S1d–e), the efficiency of VIGS in *N. cataria* remained low. Moreover, the silenced phenotype (photobleaching in the case of *ChlH*) would often remain in tissues near the site of infection, only occasionally spreading to new growth (Fig. 2a). We addressed these issues using a dual knockdown system as reported in (Yamamoto et al. 2021). In this approach, a visual marker, such as *ChlH*, is used to visually indicate which areas of tissue are affected by VIGS. This enables precise identification and excision of tissues that have been successfully silenced (Fig. 2b). We first tested the effect of silencing on the total amount of nepetalactone content in *N. cataria*. Compared to infection with an empty vector, infection with a vector targeting only *ChlH* did not alter the total nepetalactone content (Fig. 1). Examination of photobleached tissue in which *ChlH* had been silenced had a nepetalactone profile that was not significantly different compared to the empty vector infection. All interpretations of silenced genes were made relative to leaf tissue in which *ChlH* had been silenced (referred to as the control).

We selected the first step of the iridoid biosynthesis pathway, GES, as a positive control in which to test the efficacy of VIGS in perturbing the nepetalactone biosynthetic pathway. We measured the nepetalactone content when *ChlH* was silenced on its own (*chlh*), when GES was silenced on its own (*ges*), and from the photobleached sections of tissue when *ChlH* and GES were silenced simultaneously (*chlh-ges*) (Suppl. Fig. S1c). While there is no statistically significant reduction of nepetalactone content between *chlh* and *ges* tissue, *chlh-ges* samples displayed a statistically significant decrease in total nepetalactone content compared to *chlh* samples. These results validated the use of the visual marker system to study the in vivo activity of iridoid biosynthetic genes.

### VIGS of biosynthetic genes *ISY*, *MLPL*, and *NEPS*

**Fig. 3:**
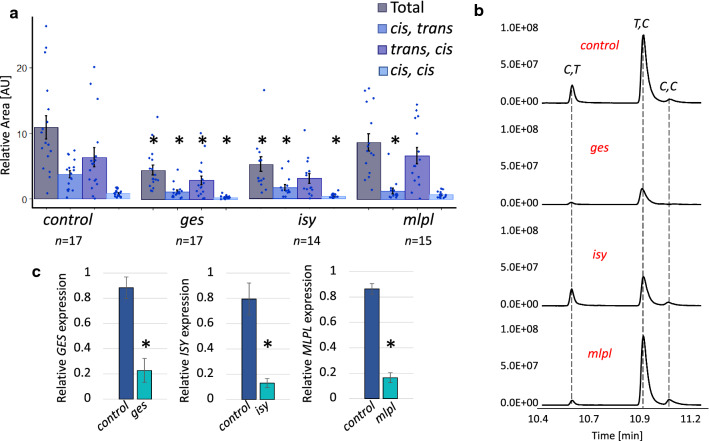
GC–MS analysis and gene expression analysis of VIGS samples for *ges*, *isy*, and *mlpl*. **a** Total normalized nepetalactone content for each gene silencing experiment. Normalized nepetalactone content for each isomer is calculated by dividing the isomer peak area by the internal standard (50 μM camphor) peak area. Total nepetalactone content is the sum of each relative isomer content. The bars indicate the average content of the sum. Each point is an individual sample. Error bars are the standard error (control *n* = 17; *ges n* = 17; *isy n* = 14; *mlpl n* = 15). An asterisk above a bracket indicates a *t* test *p* value < 0.05. AU indicates arbitrary units. **b** Representative GC–MS traces of the control (VIGS targeting ChlH), *ges, isy*,* and mlpl.* X-axis is retention time in minutes. IS, internal standard (50 μM camphor); *C,T*, (*S*)*-cis,trans* nepetalactone; *T,C*, (*S*)*-trans,cis* nepetalactone; *C,C*, (*S*)*-cis,cis* nepetalactone. **c** Relative gene expression of each targeted gene relative to *UBI9* expression in the control and each experimental tissue as measured by qPCR. *Y*-axis is relative gene expression calculated by $2^{{ - \Delta \Delta C_{{\text{t}}} }}$. Error bars are the standard error (all *n* = 3). An asterisk above a bracket indicates a *t* test *p* value < 0.05

We then silenced *ISY* and *MLPL* (Sherden et al. 2018; Lichman et al. 2019, 2020). To test the effect of the targeted genes on the pathway, we measured the total nepetalactone content as well as the amount of individual isomers using GC–MS analysis (Fig. 3a, b). As described above, we found that silencing *GES* significantly decreased the overall nepetalactone content and each individual isomer in affected tissue as compared to the control (*chlh*). Silencing of *ISY* led to a decrease in overall nepetalactone content, and specifically decreases in the isomers (*S*)-*cis,trans* and (S)-*cis,cis* nepetalactone, but not (*S*)-*trans,cis* nepetalactone, the major nepetalactone isomer present in our cultivar. *MLPL* silencing led to the expected decrease in (*S*)-*cis,trans* nepetalactone content, without affecting the ratio of the other two isomers nor resulting in a significant decrease in total nepetalactone content. Gene expression of the knockdown tissues were measured via qPCR analysis. Compared to *chlh* samples, *ges*, *isy*, and *mlpl* samples showed a significant decrease in gene expression, but not a complete silencing of each gene.

Silencing of individual NEPS proved problematic. As previously reported (Lichman et al. 2020), the *NEPS* genes have a high degree of nucleotide similarity within their gene coding region (70–87% nucleotide identity), which prohibited silencing of the individual genes using the coding regions. In an attempt to silence individual NEPS genes, the 3′ UTR region of *NEPS 1, 3, 4,* and *5* were targeted. However, the nepetalactone content did not significantly change for any of these samples, and we were not able to draw any conclusions from this data. An alternative silencing strategy must be developed for assessing the *in planta* function of the NEPS family of enzymes.

### Silencing of iridoid biosynthetic genes leads to an accumulation of derivatized precursor molecules

When a metabolic enzyme is silenced, in addition to seeing a decrease in the enzyme product, it is also possible that an upstream precursor in the pathway will accumulate. GC–MS analysis did not show the accumulation of any of the expected precursors, such as geraniol, 8-hydroxygeraniol or 8-oxo-geranial, upon knockdown of GES, ISY or MLPL. However, metabolic intermediates that accumulate in response to silencing are often derivatized by the endogenous enzymes of the plant, and geraniol is particularly susceptible to such derivatization (Dong et al. 2013, 2016; Dudley et al. 2021). To assess whether this was happening in *N. cataria*, semi-targeted metabolic analysis was carried out on the methanol extracts from the collected VIGS samples on a liquid chromatography-quadrupole time of flight mass spectrometer (LC-QTOF-MS) in negative ion mode (Dong et al. 2013, 2016; Dudley et al. 2021). All runs were then processed using the Bruker Metaboscape software (2021-B) for analysis in metabolite peak changes.

**Fig. 4:**
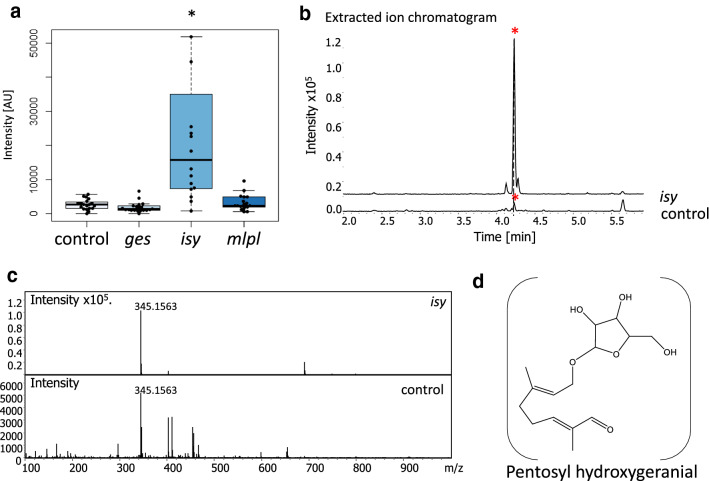
Putative derivatized geraniol-derived compounds assigned using QToF LC–MS. **a** Intensity distribution ([AU] arbitrary units) of each sample and the associated boxplot. Boxplots show median (line) and 25 and 75% quartiles. Whiskers indicate the lower and upper extremities. Asterisk over a bracket indicates a statistically significantly difference (*t* test *p* value < 0.05) (control *n* = 17; *ges n* = 17; *isy n* = 14; *mlpl n* = 15). **b** Representative extracted ion chromatograms of *m*/*z* 345.1549 of a control (*chlh*) and *isy* samples. *Y*-axis is the intensity or the area under the peak reported in arbitrary units to the × 10^5^, *Y*-axis is the retention time in minutes. An asterisk indicates the peak of interest. **c** The ms/ms of the same control and *isy* sample, indicating the *m/z* of the extracted ion. The *X*-axis is the mass to charge ratio (*m/z*) of the peak at minute 4.23. Intensity is in arbitrary units. **d** The predicted structure of the metabolite significantly upregulated in *isy* samples and a possible structure for the tentative pentosyl hydroxygeranial (formic acid adduct is not shown). The exact mass 345.1549 was calculated using the proposed molecular formula (C_15_H_24_O_6_––COOH). Difference in parts per million (Δppm) was calculated by the following formula: Δppm = 10^6^((*M*_observed_ − *M*_theoretical_)/*M*_theoretical_)

We focused on the identification of derivatized geraniol, since derivatized geraniol metabolites have been previously identified (Dong et al. 2013, 2016; Dudley et al. 2021). These metabolomic analyses revealed (Fig. 4a) an upregulation (ninefold) in *isy* tissue of a compound with a retention time of 4.23 min and a mass to charge ratio (*m/z*) of 345.1561 (Fig. 4b, c). We tentatively assigned this compound to be a formic acid adduct of a pentose conjugated hydroxygeranial (C_15_H_24_O_6_–COOH) which has an exact mass of 345.1549 (Δppm of 3.56) (Fig. 4d). Rigorous characterization was not possible due to the lack of standards available, and the amount produced was too low for isolation and characterization. Nevertheless, these observations provide indirect support that ISY is involved in this geraniol-derived pathway. Other less concentrated compounds (up to × 10^4^) were found to have significantly increase or decrease by more than ± 1.00 fold change in all genes knockdown, but were not tentatively assigned a structure (Table S2).

### Glycosylated iridoid 1,5,9 epi-deoxyloganic acid was downregulated in *ges* samples

**Fig. 5:**
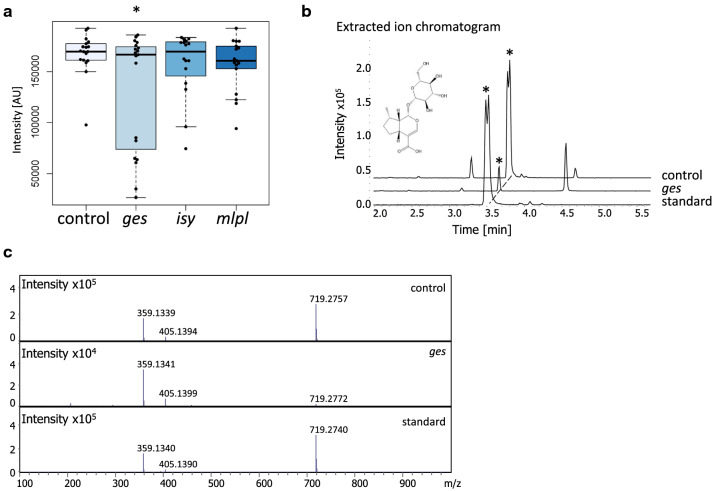
The presence of 1,5,9 epi-deoxyloganic acid in *N. cataria* samples. **a** Intensity distribution ([AU] arbitrary units) of each sample and the associated boxplot. Boxplots show median (line) and 25 and 75% quartiles. Whiskers indicate the lower and upper extremities. Asterisk over a bracket indicates a statistically significantly difference (*t* test *p* value < 0.05) (control *n* = 17; *ges n* = 17; *isy n* = 14; *mlpl n* = 15). **b** Representative extracted ion chromatograms of a control (*chlh*) and *ges* and 1,5,9 epi-deoxyloganic standard (Lichman et al. 2020) samples. *Y*-axis is the intensity or the area under the peak reported in arbitrary units to the × 10^5^, *Y*-axis is the retention time in minutes. An asterisk indicates the peak of interest. Graphs have been offset by 15° to allow for easier visualization. **c** The MS/MS of the standard, control and *ges* sample, indicating the *m*/*z* of the extracted ion. The *X*-axis is the mass to charge ratio (*m*/*z*) of the peak. Intensity is in arbitrary units

In addition to nepetalactone isomers, *N. cataria* is also reported to produce the glycosylated iridoid 1,5,9 epi-deoxyloganic acid (Fig. 5d) (Formisano et al. 2011; Lichman et al. 2020). Methanol extracts from the VIGS samples were analyzed by LC-QTOF-MS to probe for changes in the production of this compound in silenced tissues. From these data, the concentration of 1,5,9 epi-deoxyloganic acid was significantly down regulated by 1.27 fold in *ges* samples, though the variability in these samples was high (Fig. 5a–c). For the genes *ISY* and *MLPL*, no significant downregulation of 1,5,9 epi-deoxyloganic acid was observed.

## Discussion

Three distinct stereoisomers of the iridoid nepetalactone—(*S*)-*cis,trans*; (*S*)-*cis,cis* and (*S*)-*trans,cis*—are produced in varying ratios by many species in the *Nepeta* genus (Sherden et al. 2018; Lichman et al. 2019, 2020). The ecological function of these compounds remains unclear, though the insect repellent activity of these compounds suggests that nepetalactones may play a role in mediating plant–insect interactions (Birkett et al. 2011; Reichert et al. 2019; Melo et al. 2021; Uenoyama et al. 2021, 2022). Recently, the genes, along with the corresponding proteins, that are responsible for the biosynthesis of these nepetalactone stereoisomers have been discovered from *Nepeta cataria* and *Nepeta mussinii* (Lichman et al. 2019, 2020). Briefly, geranyl pyrophosphate (GPP) is converted to geraniol via the action of GES. Geraniol is then oxidized to 8-oxo-geranial, and then reduced by ISY to form a reactive enol. This enol is then captured by one of several cyclases that catalyse the formation of one of the three known nepetalactol diastereomers. A short-chain alcohol dehydrogenase then oxidizes each of these nepetalactol isomers to form the corresponding nepetalactone (Fig. 1).

The activity of these biosynthetic enzymes have only been validated by in vitro biochemical assays (Sherden et al. 2018; Lichman et al. 2019, 2020), and a demonstration that these genes are responsible for nepetalactone and nepetalactol biosynthesis *in planta* is lacking. Here, we report an optimized protocol for virus-induced gene silencing (VIGS) in *N. cataria* (catnip). We use this VIGS approach to validate that *GES*,* ISY*, and *MLPL* are involved in the biosynthesis of the nepetalactol stereoisomers. *N. cataria* was selected as the species in which to perform these experiments because propagation of *N. cataria* by cuttings proved to be more efficient than propagation of *N. mussinii* or germination of *N. cataria* seeds. Cuttings were also preferred as the isomer profile has the potential to vary across seedlings. Moreover, *N. cataria* produces a consistent and stable ratio of (*S*)-*cis,trans*, (*S*)-*cis,cis* and (*S*)-*trans,cis* nepetalactone stereoisomers, while *N. mussinii* produces primarily only a single stereoisomer, (*S*)-*cis,trans* nepetalactone.

Silencing of GES in *N. cataria* led to a statistically significant decrease of all nepetalactone isomers (Fig. 3a, b), which was expected since the biochemical product of GES, geraniol, has been established as a precursor for all known iridoids (Simkin et al. 2013; Kumar et al. 2015). GES has been subjected to VIGS in the plant *Catharanthus roseus,* which produces alkaloids derived from iridoids (Kumar et al. 2015). In this plant system, silencing of GES also led to a statistically significant decrease of iridoid-derived alkaloids. GES was also used in this study as proof of principle, as well as a way to assess the viability of target design (Suppl. Fig. S1c).

ISY is responsible for the conversion of 8-oxogeranial to a reactive enol intermediate that either spontaneously cyclizes to the (*S*)-*cis,trans* nepetalactol isomer, or is taken up by one of three downstream cyclases (Lichman et al. 2019). Silencing of ISY led to a statistically significant decrease of total nepetalactone content. Notably, however, the distribution of nepetalactone stereoisomers was not uniformly silenced, with the reduction of (*S*)-*cis,trans* and (*S*)-*cis,cis* isomers more significant than reduction in (*S*)-*trans,cis* isomers. It is possible that the reduced level of ISY product is channelled more efficiently to the cyclase responsible for (*S*)-*trans,cis* formation. Notably, a compound corresponding to derivatized geraniol was observed in *isy* silenced tissue (Fig. 4). This further validates the role of ISY in nepetalactone biosynthesis, as the presence of this compound suggests that iridoid precursors accumulate, and are subsequently derivatized, when *ISY* is silenced. Silencing of *ISY* in *C. roseus* had a similar effect; only modest decreases in nepetalactol-derived product were observed, but derivatized geraniol-based precursors were observed (Geu-flores et al. 2012). We expected all isomers to be reduced equally, or alternatively, to see a higher relative amount of the *cis*–*trans* isomer, which is the only isomer that can form spontaneously from 8-oxocitronellyl enol. The fact that (*S*)-*trans*–*cis* levels were not significantly affected suggests that there may be preferential access to this cyclase. Although there is no evidence to explain the mechanism behind this, it is possible that protein–protein interactions between ISY and selected cyclases may impact the relative levels of these cyclized stereoisomers.

MLPL had been shown to catalyse the conversion of the ISY product to the (*S*)*-cis,trans* nepetalactol isomer (Lichman et al. 2020). Consistent with this previously reported biochemical activity, when MLPL was silenced, the levels of (*S*)*-cis,trans* nepetalactone were significantly reduced in vivo*,* without significantly impacting the production of (*S*)*-trans,cis* and (*S*)*-cis,cis* nepetalactones.

In addition to nepetalactone, *Nepeta* also produces glycosylated iridoids (Murai et al. 1984; Formisano et al. 2011; Lichman et al. 2020), similar to those reported in other Lamiaceae plants (Venditti et al. 2016; Fu et al. 2018; Mint Evolutionary Genomics Consortium 2018; Mamadalieva et al. 2021). We could identify 1,5,9 epi-deoxyloganic acid in wild type *N. cataria* by co-elution and comparison of a fragmentation pattern with an authentic standard. The levels of this compound were significantly decreased when *GES* was silenced, but was not affected when *ISY* and *MLPL* were silenced. (Fig. 5). However, it is not clear if any of the identified cyclases (NEPS or MLPL) participate in the biosynthesis of glycosylated iridoids, particularly one with the stereochemistry of 1,5,9 epi-deoxyloganic acid. The production of 1,5,9 epi-deoxyloganic acid likely directly depends on the formation of geraniol from GES. However, it is important to note that the volatile nepetalactones are produced in trichomes (Lichman et al. 2019), which are known to sequester volatile secondary metabolites (Tissier et al. 2017; Schuurink and Tissier 2020). However, glycosylation can facilitate transport of secondary metabolites throughout the plant (Yazaki et al. 2007; Roy et al. 2016; Gani et al. 2021). Therefore, it is possible that non-silenced tissue may be contributing to the levels of 1,5,9 epi-deoxyloganic acid seen in bleached tissue with *GES* knockdown through transport mechanisms.

VIGS has limitations as a functional genomics tool. Most notably, gene silencing is nearly always incomplete, which can complicate interpretation of chemotypes. Moreover, the high sequence similarity of the *NEPS* genes prevented a detailed analysis of how these enzymes control the accumulation of different nepetalactone stereoisomers in *Nepeta*. Despite these obstacles, this *Nepeta cataria* VIGS system can be used to investigate the as yet uncharacterized regulatory and transport mechanisms behind the iridoid metabolism that are yet to be uncovered in this plant.

### Author contribution statement

LP, SEO, KY,and PS contributed to the study conception and design. Material preparation, data collection and analysis were performed by LP. LC and MS made some of the vector constructs. MK developed and supervised GC–MS analysis. KY and PS helped supervise silencing experiments. The manuscript was written by LP and SEO and all authors commented on previous versions of the manuscript. All authors read and approved the final manuscript.

## Supplementary Information

Below is the link to the electronic supplementary material.

Supplementary file1 (PDF 252 KB)

## Data Availability

All genes used in this study have been previously reported and references are provided.

## Abbreviations

GES: Geraniol synthase
ISY: Iridoid synthase
MLPL: Major-latex protein-like
VIGS: Virus-induced gene silencing
