# Supplementary material for: In vivo characterization of key iridoid biosynthesis pathway genes in catnip (Nepeta cataria)
Source: Planta. 2022 Oct 12;256(5):99. doi: 10.1007/s00425-022-04012-z (PMC9556426; doi:10.1007/s00425-022-04012-z)
Supplement: Supplementary file 1 — Supplementary file1 (PDF 252 KB) [file 425_2022_4012_MOESM1_ESM.pdf]

Supplemental Information for

In vivo characterization of key iridoid biosynthesis pathway genes in catnip (*Nepeta cataria*)

Lira Palmer, Ling Chuang, Marlen Siegmund, Maritta Kunert, Kotaro Yamamoto, Prashant Sonawane, Sarah E. O'Connor

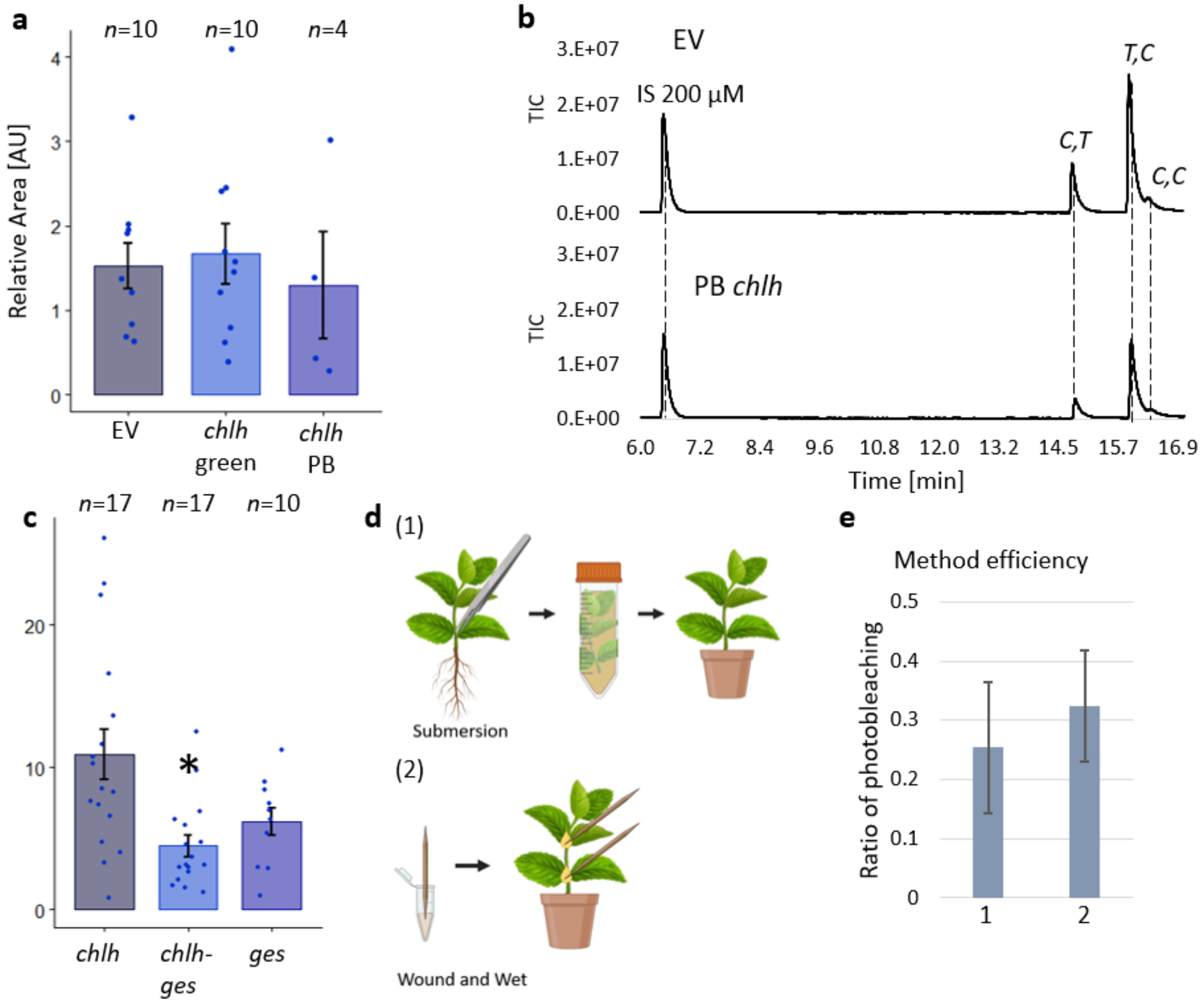

Supplemental Figure 1

a Total normalized nepetalactone content for plants infected with empty-pTRV2 vector (EV); green tissue from plants infected with pTRV2-ChlH (*chlh*); photo-bleached tissue from plants infected with pTRV2-ChlH (PB *chlh*). Normalized nepetalactone content is calculated by dividing the nepetalactone peaks areas by the internal standard (200 µM camphor). Error bars are standard error (EV *n*=10; *chlh* green *n*=10; *chlh* PB *n*=4). **b** A representative MS spectrum of EV and PB *chlh* samples. C,*T* is *(S)-cis,trans* nepetalactone, *T,C* is *(S)-trans-cis* nepetalactone and *C,C* is *(S)-cis-cis* nepetalactone. IS is internal standard (200 µM camphor). **c** Total normalized nepetalactone content for PB *chlh* tissue, photo-bleached tissue from plants infected with the double silencing vector for *chlh* and *ges* (*chlh-ges)*; green tissue from plants in which *ges* was silenced alone (*ges*). Error bars represent standard error (*chlh n*=17; *chlh-ges n*=17; *ges n*=10). Asterisk indicates statistically significant difference (t-test *P*-value <0.05). **d** Methods reported and used for infection. Method **1** (submersion) was reported in (Palmer and O'Connor, 2020);

method **2** (Wound and wet) was used in this paper. E) Ratio of photobleaching efficiency from methods 1 (submersion) and 2 (wound and wet). Ratios were calculated by counting the number of plants displaying the photobleached phenotype divided by the total number of infected plants. Error bars are standard deviation.

Supplemental Tables

Code	Gene	Sense	Sequence
LP056	NECA_E-N_VIGS_CHLH	F	TAAGGTTACCGAATTACCAATGACATGAAGGCCAC
LP057	NECA_E-N_VIGS_CHLH	R	TACCGGATCCCCATGGAGCTCACAATTTGAGGGCCAC
LP058	NECA_B-X_VIGS_GES1	F	GCCTCCATGGGGATCTCGCCTTTGTAGCGAGGTGG
LP059	NECA_B-X_VIGS_GES1	R	ATGCCCGGGCCTCGATTTGAGTACTTCGGGTCGGG
LP069	Neca_EcoR1_VIGS_GES	F	TAAGGTTACCGAATTAATCCAACGGCTGGGAATCGG
Lp070	Neca_Nco1_VIGS_GES	R	GGTGGATCCCCCATGTCGCTACAAAGGCGAGGTGC
Lp071	Neca_EcoR1_VIGS_GES	R	GCCTTCTAGAGAATTTCGCTACAAAGGCGAGGTGC
Lp072	Neca_EcoR1_VIGS_ISY	F	TAAGGTTACCGAATTATGCTGCCGCAGGCGGACACCC
Lp073	Neca_EcoR1_VIGS_ISY	R	GCCTTCTAGAGAATTGGTCTGCAAGCAGATGTGCTTC
Lp091	Neca_EcoR1_VIGS_MLP1	F	TAAGGTTACCGAATTACCAAATTATTCCCCAAGGCTT
Lp092	Neca_EcoR1_VIGS_MLP1	R	GCCTTCTAGAGAATTTGCCTTTTCAAATTCTCCTGAA
Lp131	Neca_RLK_VIGS_EcoR1	F	TAAGGTTACCGAATTGATGCTACATTTGTGCAGAGAGG
lp132	Neca_RLK_vigs_EcoR1	R	GCCTTCTAGAGAATTCCGAATGTGACTCATAC
lp228	qRT_PCR_NC_CHLH_1	F	TCAGACCACGCAAGAAGTCC
lp229	qRT_PCR_NC_CHLH_1	R	CCAAGTAGCCCACAAGCTCA
lp230	qRT_PCR_NC_CHLH_2	F	GAGCTTGTGGGCTACTTGGT
lp231	qRT_PCR_NC_CHLH_2	R	CTTCTCCACAGCAGCCTTGA
lp232	qRT_PCR_NC_GESA_1	F	CGCTTCCCAAGGACCTCAAT
lp233	qRT_PCR_NC_GESA_1	R	TCGTTGTTGTGGACTACGGG
lp234	qRT_PCR_NC_GESA_2	F	GTGTAACCGACCAAAACGCC
lp235	qRT_PCR_NC_GESA_2	R	CGAGATCATCCCAAAGGCGA
lp236	qRT_PCR_NC_ACT1_1	F	TCGTGTTGGTCCTGAAGAGC
lp237	qRT_PCR_NC_ACT1_1	R	GAGAGAACGGCCTGGATAGC
lp238	qRT_PCR_NC_ACT1_2	F	GCTATCCAGGCCGTTCTCTC
lp239	qRT_PCR_NC_ACT1_2	R	CTCACACCATCACCGGAGTC
lp240	qRT_PCR_NC_UBQ9_1	F	AGGTCGAGAGCTCGGATACT
lp241	qRT_PCR_NC_UBQ9_1	R	AGCCTTTGCTGATCTGGTGG
lp242	qRT_PCR_NC_UBQ9_2	F	GAGGATGGGAGAACCTTGGC
lp243	qRT_PCR_NC_UBQ9_2	R	CTCAAACGCAGCACCAGATG
lp248	qRT_PCR_NC_MLPL_1	F	ATGAACAAGTCCCTGATCCTGT
lp249	qRT_PCR_NC_MLPL_1	R	TGACATGTGTGGTTCATGCCA
lp250	qRT_PCR_NC_MLPL_2	F	GCAGCAGCAAAAATGGTGGA
lp251	qRT_PCR_NC_MLPL_2	R	TGACATGTGTGGTTCATGCCA
lp252	qRT_PCR_NC_RLK_1	F	GTCGGGTGAAGTCCCAGATG
lp253	qRT_PCR_NC_RLK_1	R	ATCACTTTCGGCATGAGGCA
lp254	qRT_PCR_NC_RLK_2	F	TGCCTCATGCCGAAAGTGAT
lp255	qRT_PCR_NC_RLK_2	R	TTTTTCGCAGCCCTCTCCTT
lp272	qrt_pcr_ncisy_3	F	GGTGCGGTTTTGAGGTTTCC
lp273	qrt_pcr_ncisy_3	R	GCGGCCCATATTTGATGCTC
lp274	qrt_pcr_ncisy_4	F	TTCAACGTCAGCAATGGGGA
lp275	qrt_pcr_ncisy_4	R	TTGCTGGCCTTCCTCGTATC

Supplemental Table 1. List of primers used in this study.

ges

Bucket	p-Value	Average Ratio "ges"/"chlh"	Fold Change "ges"/"chlh"	Max. Intensity
3.85 min : 360.14251 Da	0.018729	0.766	-1.274	192677
3.33 min : 568.20113 Da	0.008756	0.466	-2.093	21343
4.69 min : 348.17900 Da	0.000358	0.458	-2.127	14842

isy

Bucket	p-Value	Average Ratio "isy"/"chlh"	Fold Change "isy"/"chlh"	Max. Intensity
4.23 min : 346.16340 Da	0.005941	8.612	9.545	103129
3.97 min : 430.11185 Da	0.016313	0.344	-2.623	61906
3.91 min : 430.14828 Da	0.04477	0.251	-3.593	14039
4.54 min : 448.10203 Da	0.020802	0.464	-1.944	14011
4.02 min : 444.16400 Da	0.008218	0.393	-2.297	13504
3.75 min : 240.10021 Da	0.003275	0.43	-2.096	11595
4.20 min : 362.15717 Da	0.029325	5.736	6.357	11556

mlpl

Bucket	p-Value	Average Ratio "mlpl"/"chlh"	Fold Change "mlpl"/"chlh"	Max. Intensity
3.97 min : 430.11185 Da	0.009426	0.413	-2.7	61906
2.80 min : 342.09567 Da	0.028005	0.108	-10.347	43052
1.28 min : 190.04802 Da	0.045702	0.469	-2.38	17403
4.99 min : 186.12587 Da	0.046783	2.38	2.132	14188
3.67 min : 296.05367 Da	0.002605	0.498	-2.242	14090
4.54 min : 448.10203 Da	0.002748	0.45	-2.481	14011
3.49 min : 438.17443 Da	0.018737	0.492	-2.269	10520

Supplemental Table 2. Significantly up or downregulated metabolites identified in semi-targeted bucket correlation analyses of QToF LC-MS samples.
